# Correction to “Retinotopy of emotion: Perception of negatively valenced stimuli presented at different spatial locations as revealed by event‐related potentials”

**DOI:** 10.1002/hbm.26748

**Published:** 2024-05-23

Carretié L , Méndez‐Bértolo C , Bódalo C , et al. Retinotopy of emotion: Perception of negatively valenced stimuli presented at different spatial locations as revealed by event‐related potentials. Hum Brain Mapp. 2020; 41: 1711–1724.31860166 10.1002/hbm.24904PMC7267989

In Table 2 there are some incorrect (non‐relevant) values:

‐column 2 (PN1/SF2), file 16 (VF6/p), the third value should be “[0.839]” instead of “[0.001]”.

‐column 8 (PN2/SF3), file 4 (VF2/F), the third value should be “[0.151]” instead of “[0.455]”.

‐column 8 (PN2/SF3), file 5 (VF2/p), the third value should be “[0.700]” instead of “[0.504]”.

‐column 8 (PN2/SF3), file 6 (VF2/η2p), the three values should be “0.131, (0.047), [0.004]” instead of “0.004, (0.075), [0.012]”.

A table including these correct values is in the link to data availability cited in the paper (https://osf.io/85nu3/).

We apologize for this error.

